# Supplementary material for: A 27-country test of communicating the scientific consensus on climate change
Source: Nat Hum Behav. 2024 Aug 26;8(10):1892–905. doi: 10.1038/s41562-024-01928-2 (PMC11493676; doi:10.1038/s41562-024-01928-2)
Supplement: Supplementary file 1 — Design table, Results and Methods. [file 41562_2024_1928_MOESM1_ESM.pdf]

nature human behaviour

Supplementary information

https://doi.org/10.1038/s41562-024-01928-2

A 27-country test of communicating the scientific consensus on climate change

In the format provided by the authors and unedited

Supplementary Information

Contents

1. Design Table 1

2. Supplementary Results 6

2.1. Summary of the Frequentist Random-Effects Meta-Analytic Models 6

2.2. Percentages of Participants per Country That Underestimate the Reality Consensus and Crisis Agreement 7

2.3. Comparison Between Control and Updated Conditions 8

2.4. Three-Way Interaction Effects Between Political Ideology/Trust in Scientists, Condition, and Pre-Intervention Consensus/Agreement Perceptions 9

2.5. Main Analyses Robustness Checks (Without Demographic Covariates) 11

2.6. Exploratory Analyses of Country-Level Collectivism and Power Distance as Potential Moderators of Intervention Effectiveness 14

3. Supplementary Methods 15

3.1. Population Statistics Per Country 15

3.2. Procedure Outline 16

3.3. Updated Consensus Wording Choice 16

3.4. Pilot Study 18

3.5. Country-Specific Instrument Adaptations 20

3.6. Overview of the Analytical Approach 27

4. References 38

1. Design Table

Supplementary Table 1: Overview of RQs, Hypotheses, and the Pre-Registered Sampling, Analytic, and Interpretation Plan (Prior to Data Collection but not Submitted as a Registered Report)

Question	Hypothesis	Sampling plan	Analysis Plan	Interpretation given to different outcomes
**Q1.** Is the classic scientific consensus message **effective** compared to a control message?	**$H1_{a-e}$ (main effects: control vs. classic consensus).** Compared to the control condition, participants in the classic consensus condition will (a) perceive a higher scientific consensus that human-caused climate change is happening (controlling for pre-intervention reality consensus perceptions), (b) believe more in the reality of climate change, (c) believe more in the human causation of climate change, (d) worry more about climate change, and (e) support public action on climate change more.	Prior work suggests the most likely effect size estimate to be approximately Cohen's d = 0.50 for a) and Cohen's d = 0.10 for b-e). Our Bayes factor design analysis indicates that 10,000 participants split across three conditions warrant more than 90.9% chance of obtaining strong evidence for the alternative hypothesis based on the degree of heterogeneity for a) and more than 86.6% chance of obtaining strong evidence for the alternative hypothesis based on the degree of heterogeneity for b-e).	a) We will model the difference between conditions in post-test perceptions of scientific consensus via a Bayesian model-averaged linear mixed-effects model, adjusting for the pre-test perceptions of scientific consensus, age, gender education, and political ideology. b-e) We will model the difference between conditions for the corresponding outcomes via a Bayesian model-averaged ordinal mixed-effects model adjusting for age, gender education, and political ideology.	We will interpret the intervention's effectiveness for each outcome separately. Bayes factors larger than 1 ($BF_{10} > 1$) indicate evidence for the alternative hypothesis and Bayes factors lower than 1 ($BF_{10} < 1$) indicate evidence for the null hypothesis. We use the conventional labels when describing the strength of evidence, i.e., $1 < BF_{10} < 3$ ($1/3 < BF_{10} < 1$) as weak evidence, $3 < BF_{10} < 10$ ($1/10 < BF_{10} < 1/3$) as moderate evidence, and $BF_{10} > 10$ ($BF_{10} < 1/10$) as strong evidence.
**Q2.** Is the updated scientific consensus message **effective** compared to a control message)?	**$H2_{a-g}$ (main effects: control vs. updated consensus).** Compared to the control condition, participants in the updated consensus condition will (a) perceive a higher scientific consensus	Prior work suggests the most likely effect size estimate to be approximately Cohen's d = 0.50 for a-b) and Cohen's d = 0.10 for c-h). Our Bayes factor design analysis indicates that 10,000	a-b) We will model the difference between conditions in post-test perceptions of (a) the scientific consensus and (b) the scientific agreement via a Bayesian model-averaged linear mixed-effects	We will interpret the intervention's effectiveness for each outcome separately. Bayes factors larger than 1 ($BF_{10} > 1$) indicate

	that human-caused climate change is happening (controlling for pre-intervention reality consensus perceptions),<br>(b) perceive higher scientific agreement that climate change is a crisis (controlling for pre-intervention crisis agreement perceptions),<br>(c) believe more in the reality of climate change,<br>(d) believe more in the human causation of climate change,<br>(e) believe more that climate change constitutes a crisis,<br>(f) worry more about climate change,<br>(g) support public action on climate change more.	participants split across three conditions warrant more than 90.9% chance of obtaining strong evidence for the alternative hypothesis based on the degree of heterogeneity for a) and more than 86.6% chance of obtaining strong evidence for the alternative hypothesis based on the degree of heterogeneity for b-e).	model, adjusting for the corresponding pre-test score, age, gender education, and political ideology.<br>c-h) We will model the difference between conditions for the corresponding outcomes via a Bayesian model-averaged ordinal mixed-effects model adjusting for covariate effects age, gender education, and political ideology.	evidence for the alternative hypothesis and Bayes factors lower than 1 ($BF_{10} < 1$) indicate evidence for the null hypothesis. We use the conventional labels when describing the strength of evidence, i.e., $1 < BF_{10} < 3$ ($1/3 < BF_{10} < 1$) as weak evidence, $3 < BF_{10} < 10$ ($1/10 < BF_{10} < 1/3$) as moderate evidence, and $BF_{10} > 10$ ($BF_{10} < 1/10$) as strong evidence.
**Q3.** Is the updated scientific consensus message more **effective** than the classic consensus message?	**H3$_{a\text{-}c}$ (main effects: classic consensus vs. updated consensus).**<br>Compared to the classic consensus condition, participants in the updated consensus condition will<br>(a) believe more that climate change constitutes a crisis,<br>(b) worry more about climate change,<br>(c) support public action on climate change more.	We base the power calculations on Cohen's *d* = .10 as the smallest effect size of interest for a-c). Our Bayes factor design analysis indicates that 10,000 participants split across three conditions warrant more than 86.6% chance of obtaining strong evidence for the alternative hypothesis based on the degree of heterogeneity for a-c).	a-c) We will model the difference between conditions for the corresponding outcomes via a Bayesian model-averaged ordinal mixed-effects model adjusting for age, gender education, and political ideology.	We will interpret the intervention's effectiveness for each outcome separately. Bayes factors larger than 1 ($BF_{10} > 1$) indicate evidence for the alternative hypothesis and Bayes factors lower than 1 ($BF_{10} < 1$) indicate evidence for the null hypothesis. We use the conventional labels when describing the strength of evidence, i.e., $1 < BF_{10} < 3$ ($1/3 < BF_{10} < 1$) as weak evidence, $3 < BF_{10} < 10$ ($1/10 < BF_{10} < 1/3$) as moderate evidence, and $BF_{10} > 10$ ($BF_{10} < 1/10$) as

				strong evidence.
**Q4.** Does the effectiveness of the classic consensus message vary by subgroup?	**H4$_{a-c}$ (interaction effects: control vs. classic consensus)**<br>Controlling for pre-intervention reality consensus perceptions, the effect of the classic consensus vs. control condition on reality consensus perceptions will be moderated by:<br>a) message familiarity, such that the message will be more effective for those who report lower familiarity with the classic consensus statement, and<br>b) trust in climate scientists, such that the message will be more effective for those who report higher trust in climate scientists.<br>Controlling for pre-intervention perceptions of scientific consensus, the effect of the classic consensus vs. control condition on consensus perceptions will *not* be moderated by:<br>c) political ideology	Based on our best guess for the moderation effect size estimate, standardized regression coefficient $\beta = 0.30$, for a-c), our Bayes factor design analysis indicates that 10,000 participants split across three conditions warrant more than 99.7% chance of obtaining strong evidence for the alternative hypothesis based on the degree of heterogeneity for a-c).	We will model the interaction between the moderator and conditions by extending the Bayesian model-averaged linear mixed-effects models with the corresponding main effect of the moderator and the moderator's interaction with the condition.	We will interpret the findings for each of the three moderators separately. Bayes factors larger than 1 ($BF_{10} > 1$) indicate evidence for the alternative hypothesis and Bayes factors lower than 1 ($BF_{10} < 1$) indicate evidence for the null hypothesis. We use the conventional labels when describing the strength of evidence, i.e., $1 < BF_{10} < 3$ ($1/3 < BF_{10} < 1$) as weak evidence, $3 < BF_{10} < 10$ ($1/10 < BF_{10} < 1/3$) as moderate evidence, and $BF_{10} > 10$ ($BF_{10} < 1/10$) as strong evidence. weak evidence.

**Q5.** Does the effectiveness of the updated consensus message vary by subgroup?	We plan the following **exploratory** analyses:<br><br>Is the effect of the updated vs. control condition on reality consensus perceptions moderated by:<br>a) message familiarity (of the classic consensus message),<br>b) trust in climate scientists, and<br>c) political ideology, controlling for pre-intervention perceptions of the scientific reality consensus?<br><br>Is the effect of the updated vs. control condition on crisis agreement perceptions moderated by:<br>a) message familiarity (of the classic and the updated consensus message),<br>b) trust in climate scientists, and<br>c) political ideology, controlling for pre-intervention perceptions of the crisis agreement?<br>intervention perceptions of the crisis agreement.	No a priori power calculations.	We will model the interaction between the moderator and conditions by extending the Bayesian model-averaged linear mixed-effects models with the corresponding main effect of the moderator and the moderator's interaction with the condition.	We will interpret the findings for each of the three moderators separately. Bayes factors larger than 1 ($BF_{10} > 1$) indicate evidence for the alternative hypothesis and Bayes factors lower than 1 ($BF_{10} < 1$) indicate evidence for the null hypothesis. We use the conventional labels when describing the strength of evidence, i.e., $1 < BF_{10} < 3$ ($1/3 < BF_{10} < 1$) as weak evidence, $3 < BF_{10} < 10$ ($1/10 < BF_{10} < 1/3$) as moderate evidence, and $BF_{10} > 10$ ($BF_{10} < 1/10$) as strong evidence. weak evidence.
**Q6.** Does the effectiveness of both interventions vary by country?	We plan **exploratory** analyses to test whether the interventions' effectiveness varies by country.	We based the Bayes factor design analysis on three degrees of between-country heterogeneity in the condition differences, i.e., no heterogeneity, substantial heterogeneity equal to ½ of the effect size, and excessive heterogeneity equal to the effect size. For Q1a, Q2a-b, our Bayes factor design analysis indicates that 10,000	We will model the between-country heterogeneity in condition effects as random slopes of the condition differences via a Bayesian model-averaged linear mixed-effects model adjusting for age, gender education, and political orientation ideology for Q1a and Q2a-b and via a Bayesian model-averaged ordinal mixed-effects model adjusting for age, gender education, and political	We will look at heterogeneity separately for each model regarding Q1 and Q2. Bayes factors larger than 1 ($BF_{rf} > 1$) indicate evidence for the presence of heterogeneity and Bayes factors lower than 1 ($BF_{rf} < 1$) indicate evidence for the absence of heterogeneity. We use the conventional labels

		participants split across three conditions warrants obtaining strong evidence for the presence of excessive as well as substantial heterogeneity. For Q1b-e, Q2c-h, our Bayes factor design analysis indicates that 10,000 participants split across 3 conditions warrants 18.0% and 90.6% chance of obtaining strong evidence in the favor of the presence of heterogeneity under conditions of substantial and excessive heterogeneity, respectively.	ideology for questions Q1b-e and Q2c-h.	when describing the strength of evidence, i.e., $1 < BF_{rf} < 3$ ($1/3 < BF_{rf} < 1$) as weak evidence, $3 < BF_{rf} < 10$ ($1/10 < BF_{rf} < 1/3$) as moderate evidence, and $BF_{rf} > 10$ ($BF_{rf} < 1/10$) as strong evidence. weak evidence.

2. Supplementary Results

2.1. Summary of the Frequentist Random-Effects Meta-Analytic Models

Supplementary Table 2

Outcome	Control vs. Classic	Control vs. Updated	Classic vs. Updated
Post-intervention consensus perceptions	*d* = 0.47 [0.41, 0.52]<br>τ_c = 0.06 [0.00, 0.15]	*d* = 0.47 [0.41, 0.52]<br>τ_c = 0.05 [0.00, 0.14]	*d* = -0.01 [-0.07, 0.04]<br>τ_c = 0.08 [0.00, 0.22]
Post-intervention agreement perceptions	*d* = 0.30 [0.25, 0.35]<br>τ_c = 0.02 [0.00, 0.13]	*d* = 0.23 [0.16, 0.31]<br>τ_c = 0.15 [0.09, 0.23]	*d* = -0.12 [-0.19, -0.05]<br>τ_c = 0.14 [0.07, 0.21]
Belief in reality of climate change	*d* = 0.06 [0.01, 0.12]<br>τ_c = 0.07 [0.00, 0.20]	*d* = 0.07 [0.02, 0.12]<br>τ_c = 0.04 [0.00, 0.14]	*d* = 0.02 [-0.04, 0.07]<br>τ_c = 0.06 [0.00, 0.13]
Belief in human causation	*d* = 0.10 [0.04, 0.15]<br>τ_c = 0.06 [0.00, 0.17]	*d* = 0.09 [0.04, 0.15]<br>τ_c = 0.07 [0.00, 0.16]	*d* = 0.00 [-0.05, 0.05]<br>τ_c = 0.04 [0.00, 0.12]
Belief in crisis	*d* = 0.06 [0.01, 0.11]<br>τ_c = 0.00 [0.00, 0.12]	*d* = 0.04 [-0.01, 0.09]<br>τ_c = 0.06 [0.00, 0.14]	*d* = -0.02 [-0.07, 0.03]<br>τ_c = 0.07 [0.00, 0.14]
Climate change worry	*d* = 0.05 [-0.01, 0.10]<br>τ_c = 0.07 [0.00, 0.21]	*d* = 0.04 [-0.01, 0.08]<br>τ_c = 0.00 [0.00, 0.08]	*d* = -0.01 [-0.06, 0.04]<br>τ_c = 0.06 [0.00, 0.17]
Support for public action	d = 0.02 [-0.03, 0.08]<br>τ_c = 0.06 [0.00, 0.20]	*d* = 0.02 [-0.02, 0.07]<br>τ_c = 0.00 [0.00, 0.07]	*d* = 0.00 [-0.04, 0.05]<br>τ_c = 0.00 [0.00, 0.08]

Note. d refers to the estimated meta-analytic Cohen's *d*. The numbers in brackets indicate the 95% confidence interval. τ_c refers to the estimated meta-analytic heterogeneity across countries.

2.2. Percentages of Participants per Country That Underestimate the Reality Consensus and Crisis Agreement

Supplementary Table 3

Country	*N*	% of participants that underestimate the reality consensus	% of participants that underestimate the crisis agreement
Argentina	228	72.8 [66.5, 78.4]	49.1 [42.5, 55.8]
Australia	449	62.4 [57.7, 66.8]	30.1 [25.9, 34.6]
Austria	491	69.2 [64.9, 73.3]	31.4 [27.3, 35.7]
Brazil	468	68.8 [64.4, 72.9]	48.1 [43.5, 52.7]
Canada	399	66.4 [61.5, 71.0]	37.1 [32.4, 42.1]
China	449	83.7 [79.9, 87.0]	69.5 [65.0, 73.7]
Egypt	273	74.0 [68.3, 79.0]	42.5 [36.6, 48.6]
Georgia	417	78.4 [74.1, 82.2]	55.9 [51.0, 60.7]
Germany	634	61.7 [57.7, 65.5]	29.2 [25.7, 32.9]
India	166	75.9 [68.5, 82.0]	50.6 [42.8, 58.4]
Indonesia	395	79.2 [74.8, 83.1]	54.2 [49.1, 59.2]
Israel	431	80.7 [76.6, 84.3]	55.7 [50.8, 60.4]
Italy	434	75.3 [71.0, 79.3]	42.4 [37.7, 47.2]
Lebanon	9	77.8 [40.2, 96.1]	33.3 [9.0, 69.1]
Malta	470	74.3 [70.0, 78.1]	42.3 [37.8, 47.0]
Mexico	401	72.3 [67.6, 76.6]	38.7 [33.9, 43.6]
Netherlands	430	76.5 [72.2, 80.4]	46.7 [42.0, 51.6]
Poland	432	74.1 [69.6, 78.1]	53.7 [48.9, 58.5]
Portugal	506	74.9 [70.8, 78.6]	47.2 [42.8, 51.7]
Serbia	526	78.1 [74.3, 81.5]	56.7 [52.3, 60.9]
Singapore	187	70.1 [62.9, 76.4]	42.8 [35.6, 50.2]
Slovenia	458	76.4 [72.2, 80.2]	47.6 [43.0, 52.3]
Sweden	518	62.9 [58.6, 67.1]	38.6 [34.4, 43.0]
Tunisia	92	72.8 [62.4, 81.3]	40.2 [30.3, 51.0]
Türkiye	480	78.8 [74.8, 82.3]	41.7 [37.2, 46.2]
UK	422	64.5 [59.7, 69.0]	33.2 [28.7, 37.9]
USA	362	57.5 [52.2, 62.6]	39.8 [34.7, 45.0]

Note. The numbers in brackets indicate the 95% confidence interval.

2.3. Comparison Between Control and Updated Conditions

We examined the effects of the updated consensus message on reality consensus and crisis agreement perceptions, climate change beliefs (reality and human-causation), worry, and policy support compared to the control condition.
Besides the results described in the main text, we find that the effects on climate change beliefs ($H2_c$ and $H2_d$), worry ($H2_f$), and support for public action ($H2_g$) of the updated condition are consistent with those of the classic condition.

In terms of climate change beliefs ($H2_c$ and $H2_d$), we find extremely strong (BF_{+0} = 131.63) and very strong (BF_{+0} = 69.86) support that people believe more in climate change and human activity as its main cause after being exposed to the updated compared to the control message. Both intervention effects are small (reality: Cohen's d = 0.07, 95% CI [0.02, 0.12]; human-causation: Cohen's d = 0.09 [0.04, 0.15]), but consistent across the 27 studied countries (reality: $BF_{10} = 3.45\text{x}10^{-6}$, τ_c = 0.04, 95% CI [0.00, 0.14]; human causation: $BF_{10} = 5.45\text{x}10^{-5}$, τ_c = 0.07, 95% CI [0.00, 0.16]). However, there is only weak evidence for an effect of the updated scientific consensus intervention on climate change worry ($H2_f$) across countries (BF_{+0} = 2.05; Cohen's d = 0.04, 95% CI [-0.01, 0.08]; between-country heterogeneity: $BF_{10} = 2.53\text{x}10^{-11}$, τ_c = 0 [0, 0.08]). In contrast to $H2_g$, the evidence for an effect of the updated scientific consensus message on climate policy support is consistently weak across the 27 countries (BF_{+0} = 0.65, Cohen's d = 0.02 [-0.02, 0.07]; between-country heterogeneity: $BF_{10} = 1.83\text{x}10^{-9}$, τ_c = 0, 95% CI [0, 0.07]).

We also explored effects on confidence in agreement perceptions. We find extremely strong exploratory evidence that participants are more confident in their agreement perceptions after seeing the updated compared to the control message (not preregistered; $BF_{10} = 8.42\times10^{14}$; Cohen's d = 0.76; 95% CI [0.70, 0.79]; between-country heterogeneity: BF_{10} = 5.81, τ_c = 0.10, 95% CI [0.03, 0.20]).

2.4. Three-Way Interaction Effects Between Political Ideology/Trust in Scientists, Condition, and Pre-Intervention Consensus/Agreement Perceptions

Supplementary Fig. 1A-D

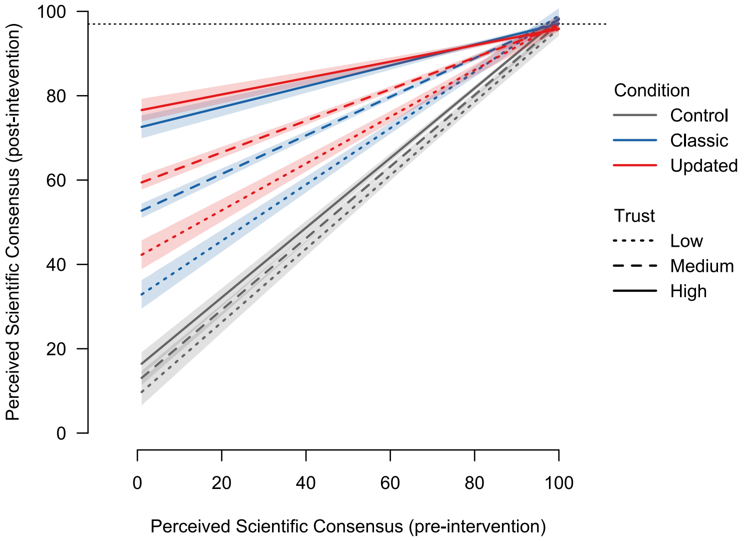

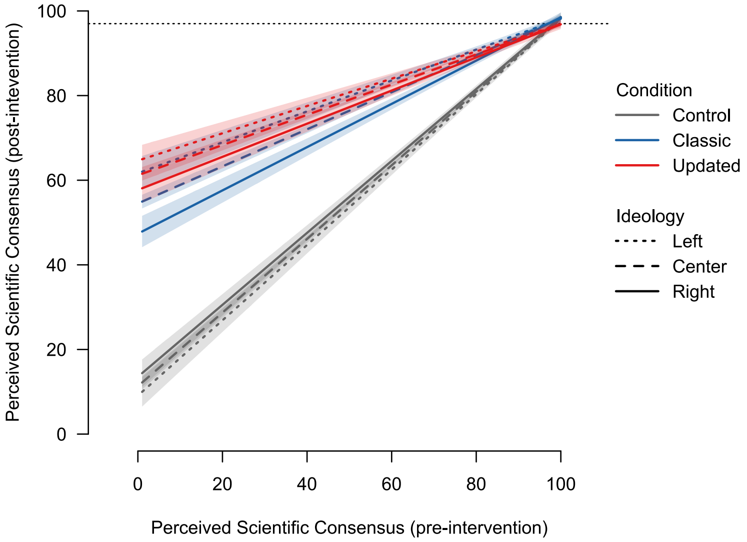

Note. The lines represent means and the shaded bands represent the 95% confidence intervals.

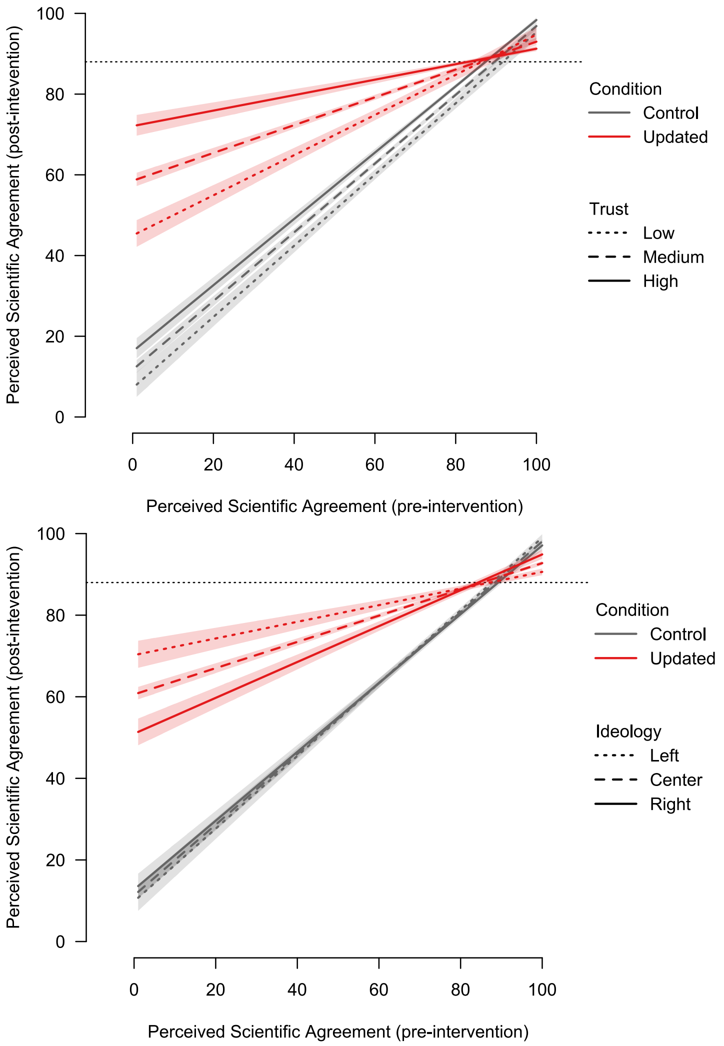

Note. The lines represent means and the shaded bands represent the 95% confidence intervals.

2.5. Main Analyses Robustness Checks (Without Demographic Covariates)

Supplementary Table 4

Conditions compared	Hypothesis #	Hypothesis summary	BF_10	BF_rf	Evidence in favor of the hypothesis	Evidence consistency with main analysis
Classic vs. control	H1a	Reality consensus perception is higher in the classic condition	1.99×10^{12}	1.58×10^{6}	extreme for	YES
	H1b	Belief in the reality of climate change is higher in the classic condition	25.98	5.66×10^{-7}	strong for	YES
	H1c	Belief in the human causation of climate change is higher in the classic condition	468.37	3.00×10^{-5}	extreme for	YES
	H1d	Climate change worry is higher in the classic condition	2.82	1.91×10^{-8}	weak/undecided for	Consistent direction, albeit less support
	H1e	Support for public action is higher in the classic condition	0.61	3.70×10^{-9}	weak/undecided against	YES
Updated vs. control	H2a	Reality consensus perception is higher in the updated condition	1.79×10^{12}	1,405.63	extreme for	YES
	H2b	Crisis agreement perception is higher in the updated condition	1.40×10^{5}	1.40×10^{8}	extreme for	YES
	H2c	Belief in the reality of climate change is higher in the updated condition	121.30	3.53×10^{-6}	extreme for	YES
	H2d	Belief in the human causation of climate change is higher in the updated condition	95.33	6.75×10^{-5}	very strong for	YES
	H2e	Belief in climate change as a crisis is higher in the updated condition	1.49	5.93×10^{-8}	weak/undecided for	YES
	H2f	Climate change worry is higher in the updated condition	2.06	1.11×10^{-10}	weak/undecided for	YES
	H2g	Support for public action is higher in the updated condition	0.68	2.19×10^{-9}	weak/undecided against	YES
Classic vs. updated	H3a	Belief in climate change as a crisis is higher in the updated condition	0.09	1.43×10^{-7}	strong against	YES

	H3b	Climate change worry is higher in the updated condition	0.14	$1.44\ x10^{-8}$	moderate against	YES
	H3c	Support for public action is higher in the updated condition	0.24	$9.78\ x10^{-11}$	moderate against	YES
Moderators: classic vs. control	H4a	The classic message is more effective for people with lower reality consensus message familiarity	$1.86\ x10^{15}$		extreme for	YES
	H4b	The classic message is more effective for people with higher trust in climate scientists	0		extreme against	YES
	H4c	The classic message is equally effective for people across the political ideology spectrum	1.67		weak/undecided for	YES
Moderators: updated vs. control (exploratory)	Q5	Is the effectiveness of the updated message in increasing reality consensus perceptions moderated by reality consensus message familiarity?	$1.03x10^{22}$		extreme for	YES
		Is the effectiveness of the updated message in increasing reality consensus perceptions moderated by trust in climate scientists?	$4.35x10^{50}$		extreme for	YES
		Is the effectiveness of the updated message in increasing reality consensus perceptions moderated by political ideology?	2.75×10^{5}		extreme for	YES
		Is the effectiveness of the updated message in increasing crisis agreement perceptions moderated by crisis agreement message familiarity?	$6.49x10^{18}$		extreme for	YES
		Is the effectiveness of the updated message in increasing crisis agreement perceptions moderated by trust in scientists?	$5.84x10^{90}$		extreme for	YES
		Is the effectiveness of the updated message in increasing crisis agreement	$4.99x10^{16}$		extreme for	YES

	perceptions moderated by political ideology?

2.6. Exploratory Analyses of Country-Level Collectivism and Power Distance as Potential Moderators of Intervention Effectiveness

Analogous with the confirmatory moderation analyses, we used Bayesian mixed-effects linear regressions, with participants (level 1) nested in countries (level 2) and controlling for relevant demographic characteristics (i.e. age, gender, university degree, and political ideology). All model configurations were identical to confirmatory models.

For the classic consensus message, we find moderate and strong evidence against any moderation of the effect on consensus perceptions by individualism-collectivism (BF_{10} = 0.20; *b* = -0.05, 95% CI [-0.11, 0.01]) and power distance (BF_{10} = 0.08; *b* = 0.03, 95% CI [-0.03, 0.10]) respectively. This was also the case for the updated consensus message: we find moderate evidence against a moderation by individualism-collectivism (BF_{10} = 0.18, *b* = -0.04, 95% CI [-0.10, 0.02]) and power distance (BF_{10} = 0.16, *b* = 0.05, 95% CI [-0.01, 0.11]) on consensus perceptions. For agreement perceptions changes in the updated (vs. control) condition, the results are inconclusive, with weak evidence against a moderation by individualism-collectivism (BF_{10} = 0.35, *b* = -0.07, 95% CI [-0.15, 0.02]) and weak evidence for a moderation by power distance (BF_{10} = 1.53, *b* = 0.08, 95% CI [0.02, 0.15]).

3. Supplementary Methods

3.1. Population Statistics Per Country

Supplementary Table 5

Country	Median (or mean) age[1]	% female[2]	% aged 25-64 years with post-secondary education attainment[3]	% living in urban area[4]
Argentina	31.90 (2023)[1]	50.50 (2022)[2]	24.80 (2021)[5]	92.00 (2022)[4]
Australia	37.50 (2023)[1]	50.30 (2022)[2]	51.60 (2020)[3]	86.00 (2022)[4]
Austria	43.10 (2023)[1]	50.80 (2022)[2]	32.10 (2019)[3]	59.00 (2022)[4]
Brazil	33.60 (2023)[1]	50.90 (2022)[2]	19.20 (2022)[6]	88.00 (2022)[4]
Canada	40.60 (2023)[1]	50.30 (2022)[2]	62.00 (2021)[7]	82.00 (2022)[4]
China	39.00 (2023)[1]	49.00 (2022)[2]	37.00 (2020)[7]	64.00 (2022)[4]
Egypt	24.20 (2023)[1]	49.40 (2022)[2]	13.00 (2017)[3]	43.00 (2022)[4]
Georgia	38.00 (2023)[8]	52.00 (2023)[8]	57.00 (2019)[3]	60.00(2022)[4]
Germany	44.80 (2022)[1]	50.70 (2022)[2]	39.50 (2020)[3]	78.00 (2022)[4]
India	28.20 (2023)[1]	48.40 (2022)[2]	13.40 (2020)[3]	36.00 (2022)[4]
Indonesia	29.60 (2022)[1]	49.70 (2022)[2]	13.20 (2021)[3]	58.00 (2022)[4]
Israel	29.10 (2023)[1]	50.10 (2022)[2]	49.7 (2021)[7]	93.00 (2022)[4]
Italy	47.70 (2023)[1]	51.20 (2022)[2]	20.00 (2021)[5]	72.00 (2022)[4]
Lebanon	28.80 (2023)[1]	51.50 (2022)[2]	NA	89.00 (2022)[4]
Malta	39.80 (2023)[1]	47.90 (2022)[2]	32.70 (2020)[3]	95.00 (2022)[4]
Mexico	29.80 (2023)[1]	51.20(2022)[2]	20.50 (2021)[5]	81.02 (2022)[9]
Netherlands	42.50(2023)[10]	50.28 (2023)[10]	43.10 (2021)[10]	93.00 (2022)[4]
Poland	40.00 (2023)[1]	51.60 (2022)[2]	31.50 (2020)[3]	60.00 (2022)[4]
Portugal	45.80 (2023)[1]	52.80 (2022)[2]	23.10 (2020)[3]	67.00 (2022)[4]
Serbia	43.20 (2023)[1]	52.10 (2022)[2]	22.60 (2019)[3]	57.00 (2022)[4]
Singapore	42.30 (2022)[1]	47.70 (2022)[2]	58.30 (2020)[3]	100.00 (2022)[4]
Slovenia	44.00 (2023)[11]	51.10 (2022)[11]	20.00 (2022)[11]	56.00 (2022)[4]
Sweden	40.79 (2022)[12]	49.60 (2022)[2]	45.00 (2021)[12]	88.00(2022)[4]
Tunisia	32.30 (2023)[1]	50.60(2022)[2]	15.20 (2016)[3]	70.00 (2022)[4]
Türkiye	31.80 (2023)[1]	49.90 (2022)[2]	23.90 (2022)[13]	77.00 (2022)[4]
United Kingdom	40.10 (2023)[1]	50.60 (2022)[2]	50.10 (2021)[7]	84.00 (2022)[4]
United States	38.10 (2023)[1]	50.50 (2022)[2]	50.30 (2021)[7]	83.00 (2022)[4]

Note. References:

[1] United Nations Data Portal (2023). *Median Age of Population.* https://population.un.org/dataportal/data/indicators/67/locations/32/start/1990/end/2023/table/pivotbylocation

[2] The World Bank (2023). *Population, female (% of total population).* https://data.worldbank.org/indicator/SP.POP.TOTL.FE.ZS.

[3] The World Bank (2023). *Educational Attainment, at least completed post-secondary, population 25+.* https://data.worldbank.org/indicator/SE.SEC.CUAT.PO.ZS?end=2020&most_recent_value_desc=true&start=1970&view=chart

[4] The World Bank (2023). Urban population (% of total population). https://data.worldbank.org/indicator/SP.URB.TOTL.IN.ZS?end=2022&start=1960&view=chart

[5] OECD (2023). *Adult education level.* https://data.oecd.org/eduatt/adult-education-level.htm#indicator-chart
[6] IBG Educa (2023). *Conheça o Brasil - População Educação.* https://educa.ibge.gov.br/jovens/conheca-o-brasil/populacao/18317-educacao.html#:~:text=N%C3%ADvel%20de%20Instru%C3%A7%C3%A3o&text=No%20Brasil%2C%2053%2C2%25,%2C2%25%20no%20mesmo%20ano.
[7] OECD GPS Education (2023). *Education at a Glance 2022 (EAG 2022): Highlights.* https://gpseducation.oecd.org/IndicatorExplorer?plotter=h5&query=22
[8] GEOSTAT (2023). *Population.* https://www.geostat.ge/en/modules/categories/41/population
[9] Population Reference Bureau (2023). *Percent of Population Living in Urban Areas.* https://www.prb.org/international/indicator/urban/snapshot
[10] Centraal Bureau voor de Statistiek (2023). https://www.cbs.nl/
[11] Republika Slovenija Statistični Urad (2023). https://www.stat.si/statweb
[12] Statistikmyndigheten (2023). https://www.scb.se/
[13] Turkish Statistical Institute (2023). *National Education Statistics, 2022.* https://data.tuik.gov.tr/Bulten/Index?p=Ulusal-Egitim-Istatistikleri-2022-49756

3.2. Procedure Outline

Supplementary Figure 2

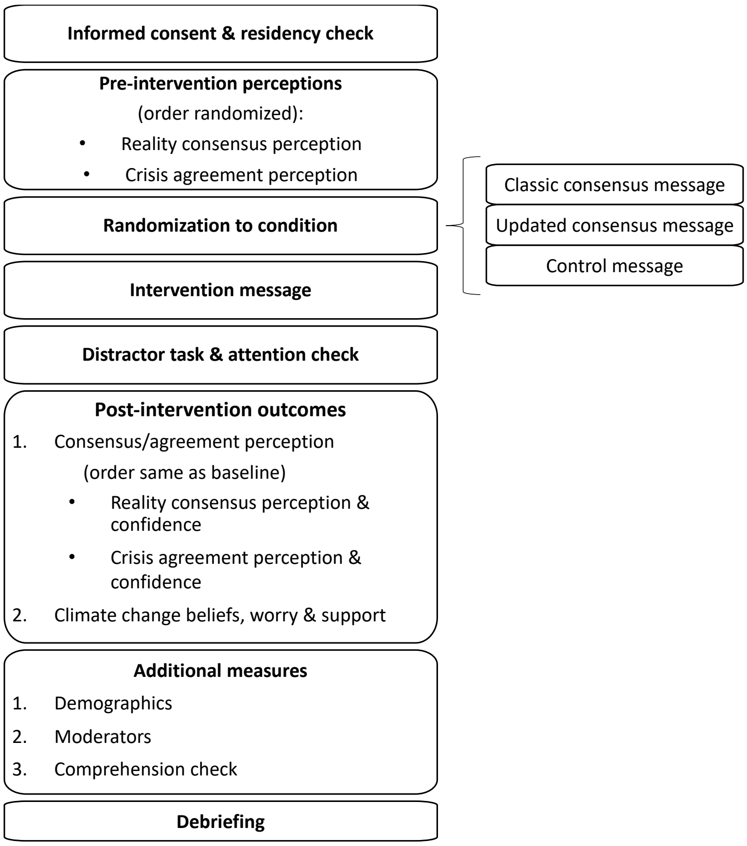

Note. This is a depiction of the order (from top to bottom) in which study materials (i.e., measures and the messaging intervention) were shown to all participants.

3.3. Updated Consensus Wording Choice

The wording of the scientific crisis agreement message is based on the *Nature* survey of IPCC 6 authors[1]. Albeit this quantification of the crisis agreement among scientists has not been obtained in the same way the 97-99% consensus on the reality of climate change (i.e., through a review of peer-reviewed climate science research), we opted for a numerical quantifier of the agreement on the urgency of climate change to maintain consistency between the format of the two statements. We did so because a statement communicating

unquantified high scientific confidence in climate change risks and the need for immediate action (e.g., see IPCC Working Group II headline statements[21]) might not be as persuasive compared to the explicitly quantified consensus on the reality of climate change. This is especially critical when the two statements are shown as one message and therefore open to direct comparisons by participants. This is further corroborated by findings showing the numeric (i.e., more precise), as opposed to verbal (i.e., less precise), message on the scientific consensus on the reality of climate change is generally more effective in increasing perceived consensus[2].

3.4. Pilot Study

We conducted a pilot study among 395 US-American participants to test the wording of the updated consensus message—a combined message that communicated both the scientific agreement on climate change as a crisis (i.e., '88% of climate scientists agree that climate change constitutes a crisis') and the scientific consensus on the reality of climate change (i.e., 97% of climate scientists agree that human-caused climate change is happening'). We tested this message against two other messages: a message that only communicated the scientific agreement that climate change constitutes a crisis and the 'classic' consensus message. The pilot also served to derive estimates for the data simulation as part of the Bayesian design analysis (i.e., the correlation between pre-post-intervention measurements; how the upper bound affects the residual variance in the intervention conditions; how the interventions influence the pre-post-intervention correlations; see Analysis plan for more information).

Procedure

In this pilot study, we used the same procedure and materials as described in this Registered Report, except that the pilot was run on a paid US sample on Prolific (February 10, 2023). Due to resource constraints, we merged these data with a control condition (i.e., '97% of dentists recommend brushing your teeth twice a day') data from a previous pilot study conducted on Prolific with a US sample (February 2-3, 2023).

Participants

After filtering and excluding inattentive participants, the final sample included 395 US-American participants (*M* = 38.01 years, *SD* = 13.55; 47.34% female). Most of them were employed full-time (52.15%), White (71.29%), and had a slightly below average, average, or slightly above average income.

Results

In Supplementary Table 5, we report descriptive statistics, including means and standard deviations, of all outcomes. The results of the pilot study (*N* = 395) provide initial, descriptive evidence that the updated message that emphasizes both the scientific consensus on the reality of climate change and agreement on climate change as a crisis may increase all outcomes compared to the control condition, as well as boost worry and support for public action more than the classic scientific consensus message. On the other hand, the updated message that communicates only the scientific crisis agreement seems to reduce belief in climate change and its human causation, worry, as well as support for action compared to all other conditions. This suggests that communicating scientists' perceptions of climate change as a crisis, in the absence of the 97% consensus on the reality of climate change, is likely not an effective message for public climate change communication efforts. Based on these initial results, we selected the combined message for the main confirmatory study.

Supplementary Table 6: Descriptive Statistics for Each Outcome per Experimental Condition

Condition	*n*	Scientific Reality Consensus (pre)	Scientific Crisis Agreement (pre)	Scientific Reality Consensus (post)	Scientific Crisis Agreement (post)	Belief in Reality	Belief in Human Causation	Belief in Crisis	Worry	Support for Action
Control	126	83.25 (17.97)	NA (NA)	83.67 (18.15)	NA (NA)	6.07 (1.51)	5.54 (1.57)	NA (NA)	5.02 (1.88)	5.76 (1.37)
Classic	87	83.59 (17.20)	84.05 (17.11)	93.87 (9.84)	90.98 (13.49)	6.34 (1.33)	5.80 (1.52)	5.77 (1.73)	5.22 (1.86)	5.90 (1.30)
Crisis only	94	80.19 (18.01)	79.72 (18.52)	85.24 (13.42)	86.36 (6.59)	5.91 (1.45)	5.24 (1.58)	5.36 (1.84)	4.66 (1.92)	5.51 (1.60)
Updated	88	85.42 (16.05)	85.06 (16.10)	94.16 (6.67)	88.22 (6.00)	6.33 (1.42)	5.83 (1.25)	5.92 (1.56)	5.32 (1.69)	6.10 (1.29)

Note. Control = ‘97% of dentists recommend brushing your teeth twice per day’; Classic = ‘97% of climate scientists agree that human-caused climate change is happening’; Crisis only = '88% of climate scientists agree that climate change is a crisis’; Updated = Classic + Crisis. As the control condition is from another pilot study, scientific agreement pre- and post-intervention, as well as belief in climate change as a crisis are missing.

3.5. Country-Specific Instrument Adaptations

The following table describes all major contextual considerations and adaptations to the survey instrument. All adaptations were approved by the two lead authors. Example adaptations include clarifying what is meant with left and right in the political ideology item, adding the English term "climate change" in parenthesis after the term in the local language, and adding "you yourself/you personally" to clarify that the item asks about an individual's opinion.

Supplementary Table 7

Country	Adaptation #	Item(s)	What did you adapt?	Reason for adaptation
Argentina	#1	Education	Separated preparatory programs from vocational school	Preparatory programs last only 1 year, whereas vocational school in Argentina is integrated within universities and can extend up to seven years
	#2	Attention check	Changed "somewhat agree" to *"I somewhat agree"*, along with adding first-person pronouns to corresponding prompts	Grammatical clarification
Australia	#1	Ethnicity	Changed to "Please describe which best describes your ancestry"	The adapted wording is more in line with the language used in the Australian Census, from which the ethnicity classification is derived.
Austria	#1	All items including "believe" (CC_real, CC_human, CC_crisis)	Changed believe to "in my opinion"	The word "believe" is in German-speaking countries often understood to refer to belief in God.
Brazil	#1	Education	Added "completed" instead of "received"	"Completed education" is a more commonly used wording.
	#2	Cons_pre	Added "scientists who research the climate (climate scientists)" instead of only "climate scientists".	To ensure that participants understand the term "climate scientists" in this and all following items.
Canada	#1	Education	"College" scored as 1 (university degree)	The US base version had no option that describes a trade program. Some college degrees typically enable individuals to earn a one or two-year diploma or certificate,

				while universities typically award three or four-year bachelor's degrees (see Ref).
	#2	Region	Changed “state” to “province or territory”	More suitable for the Canadian context
	#3	Education	Changed “some college” to “college diploma”<br><br>Changed “University degree (BA, BSc, BEng, etc.)”<br><br>Changed “4-year degree” to “Graduate degree (MA, MSc, MEng, etc.)”<br><br>Changed “Professional degree” to “Professional degree (LLM, MBA, JD, etc.)”<br><br>Changed “Doctorate” to “Doctorate (MD, PhD, etc.)”	Adaptations were made based on Canadian survey standards (see Ref) from the Canadian Institute of Health Information and personal email exchange with Census Canada.
	#4	Ethnicity	All ethnicities changed to include examples and to expand on local populations.	Adaptations were made based on Canadian survey standards (see Ref) from the Canadian Institute of Health Information and personal email exchange with Census Canada.
China	#1	CC_intro	Added the English term “climate change” in parenthesis	Clarification of the term
	#2	Education	“Professional degree” scored as 0 (no university degree)	The two-year profession training schools in China are considered further education, but not university.
Egypt	#1	All items including “climate change is happening” (Cons_pre, Classic, Updated, Cons_post, CC_real, CC_human, Fam_cons, Comprehension)	Changed “happening” to “actually happening”	The direct translation of “happening” does not convey the intended meaning in Egyptian Arabic.

	#2	All items including "human-caused climate change"	Changed "human-caused climate change" to "Climate changes caused by human activities"	The term "human-caused" is difficult to translate explicitly in Egyptian Arabic.
	#3	General	Changed all words in uppercase to underlined	There is no uppercase in Arabic.
	#4	Education	Categories were set to:<br>"Did not attend school", "Primary education",<br>"Preparatory education",<br>"Secondary education" (e.g., high school),<br>"Higher education" (e.g., university degree or higher education diploma),<br>"Postgraduate education" (e.g., master's degree or equivalent), and<br>"Doctorate".	The adapted categories better align with the Egyptian education system.
	#5	Ideology	Changed "Where would you place yourself on this scale" to "Show your political orientation on this spectrum"	Provided better clarification to Egyptians
	#6	Ethnicity	Condensed the list of choices to "Egyptian, Other, Born outside Egypt, Prefer not to answer"	More suitable for Egypt's context
Georgia	#1	Education	"Professional education" scored as 0 (no university degree)	Changed bachelor's degree to *bachelor's or equivalent* as per [55,56,66]. Professional degree therefore implies that individuals have a degree that is not equivalent to *university education*.
Germany	#1	All items including "believe" (CC_real, CC_human, CC_crisis)	Changed believe to "in my opinion"	The word "believe" is in German-speaking countries, often understood to refer to belief in God
	#2	Education	Changed the categories to "No degree", "Compulsory education", "Apprenticeship", "Bachelor's", and "Master's"	The adapted categories better align with the German education system.

	#3	Ethnicity	Added “I consider myself (a)...” and deleted the first seven categories and added “German citizen (no migration background)” and “German citizen (with migration background)”	More suitable considering historical events
			Changed “Born outside of Germany” to “No German citizen”.	More commonly used
			Deleted “More than one of the above”	More suitable considering historical events
India	#1	Education	Changed “high school” to “Intermediate School”	“Intermediate school” is more commonly used to describe completing the 12th grade.
			Changed “Professional Degree” to “Master’s Degree”	“Professional” can be used when referring to technical training and undergraduate education.
Indonesia	#1	Info	Added English term “screenshot” in parenthesis	Clarification
	#2	CC_intro	Added English term “climate change” in parenthesis	Clarification
	#3	Ideology	Added explanation “left refers to communist/ socialist political ideology”, and “right refers to nationalist/ liberal political ideology”	Adaptation to Indonesian political context
	#4	Ethnicity	Changed to “Please choose what best fits your cultural background”	Adaptation to Indonesian context
Israel	#1	Education	Changed 2-year degree” to “Certificate studies”	Adaptation to cultural context
			“Certificate studies” scored 0 (no university degree). Anything below this level of education was scored 0, and anything above was scored 1.	Certificate programs typically provide specialized job training instead of theoretical comprehension commonly found in university education.

Italy	#1	All items including “you” (CC_real, CC_human, CC_crisis, CC_concern)	Changed “you” to “personally”	Writing the subject explicitly is not common in Italian grammar
	#2	Education	Changed “Professional degree” to “Technical degree” (scored 0, no university degree).	Professional degrees correspond to university degrees in Italy, but there are also two-year technical degrees classified as further education that do not fall in the university category.
	#3	General	Translated “popular media topics” with “temi di attualità”	“Attualità” covers both media and popular
Lebanon	#1	All items and messages including “climate change is happening”	Changed “climate change is happening” to “climate change is actually happening”.	The sentence would otherwise have a different meaning.
Maltese islands	#1	Climate change introduction	Added the English term “climate change” in parenthesis	Clarification
	#2	Education	“Higher diploma” scored as 1 (university degree)	At UoM, you would need to achieve a Bachelor's degree (BA or BSc) before pursuing the higher diploma. These degrees are typically more general rather than specific.
Mexico			None	
Netherlands			None	
Poland	#1	All items including “you” (CC_real, CC_human, CC_crisis and CC_concern)	Changed “you” to “personally”	Polish grammar
	#2	CC_real and CC_crisis	Changed “strongly” to “definitely”	Better linguistic flow
	#3	Education	6 instead of 7 categories (No professional degree)	“Professional degree” is treated as “Master’s degree”.
			Removed the category “college” and added “higher vocational education”.	The “college” category is not relevant for the Polish educational system.
Portugal	#1	Info	Added a paragraph on “local responsibility for data collection”	Due to local ethics committee approval
	#2	Residency	Added “for at least one year”	To prevent participation from foreign nationals on shorter

				exchange programs (due to data collection being planned from a university mailing list).
	#3	Education	Replaced “completed” with “received”	The more commonly used wording
	#4	Cons_pre	Added “scientists who research the climate (climate scientists)” instead of just “climate scientists”	To ensure that participants understand the term “climate scientists” in this item and all following items.
Serbia	#1	All items including “you” (CC_real, CC_human, CC_crisis, and CC_concern)	Added “personally” to the item	To emphasize personal opinion, because the formal pronoun for the second person singular is the same as second person plural (i.e., *Vi*).
Singapore	#1	CC_intro	Added the English term “climate change” in parenthesis	Aiding multilingual local residents to better understand the context
	#2	Ideology	Added “progressive” and “conservative” in parenthesis	Clarification with the cultural context
	#3	Ethnicity	Added “I consider myself…” before choice selection	Clarification
Slovenia	#1	All items including “you” (CC_real, CC_human, CC_crisis, and CC_concern)	Added “you yourself” instead of only “you”	Formal and plural grammar use the same word for “you”
	#2	Ethnicity	Added “I consider myself…”	Clarification
	#3	Cons_pre	Added “scientists who study the climate” in parenthesis	To explain the term “climate scientists”, as the term “Climate scientists” is not commonly used in Slovenian.
Sweden	#1	Attention	Changed “agree strongly” to “fully agree”	Aligns with the commonly used terminology

	#2	Ethnicity	Place of birth was used instead of ethnic group categories	Ethnic group categories are not commonly used
			Removed the last option “More than one of the above / I prefer to use: (please describe)”	All categories were mutually exclusive
Tunisia	#1	All items and messages including “climate change is happening”	Changed “climate change is happening” to “climate change is actually happening”.	The sentence would have a different meaning.
Türkiye	#1	Education	“Associate Degree” (Ön lisans) scored as 0 (no university degree).	Associate Degree is not considered a complete university degree.
UK	#1	Education	Added “A-levels, GCSE, etc.”, as an example for Secondary School	To enhance comprehension as there are multiple secondary degrees.
			Added “Vocational Training, Foundation Degrees, etc.”, as an example of College	To enhance comprehension as there are multiple college programs.
			Added “Apprenticeship” to the College Category	Apprenticeship is scored as similar to College in the UK Hierarchy.
			Added “MBA” (Master of Business Administration) as a separate category	MBA has a higher recognition in the UK compared to other graduate degrees.
	#2	Intro	Added “in the UK and multiple other countries”	To clarify that the study is conducted in the UK
US	None			

3.6. Overview of the Analytical Approach

General overview of Bayesian hypothesis testing and Bayesian model averaging

We base our analyses on Bayesian hypothesis testing and Bayesian model-averaging frameworks[3–5]. Bayesian hypothesis testing uses Bayes factors,

$$BF_{10} = \frac{p(H_1|data)}{p(H_0|data)} \Big/ \frac{p(H_1)}{p(H_0)},$$

to quantify the evidence in favor of the alternative (H_1) vs. the null (H_0) hypothesis (as a change from prior to posterior odds). Bayes factors are a continuous measure of evidence that quantifies how likely the data are under each of the hypotheses, with $BF_{10} > 1$ showing support for the alternative hypothesis and $BF_{10} < 1$ showing support for the null hypothesis[6,7]. We will use the conventional labels when referring to the degree of evidence for each hypothesis, i.e., Bayes factors between 1 and 3 (between 1 and 1/3) are considered weak evidence, Bayes factors between 3 and 10 (between 1/3 and 1/10) are considered moderate evidence, and Bayes factors larger than 10 (1/10) are considered strong evidence in favor of (against) a hypothesis[8,9].

Bayesian model-averaging extends Bayesian hypothesis testing to multi-model settings. This allows for incorporating uncertainty about the specified models into the analyses and for drawing more robust conclusions[10]. Throughout the analysis, we will specify different null and the alternative hypotheses via sets of models (H_0: $M_{01}, \ldots, M_{0A}$, H_1: $M_{11}, \ldots, M_{1B}$, respectively). In such settings, we use inclusion Bayes factors,

$$BF_{10} = \frac{\sum_{b=1}^{B} p(M_{1b}|data)}{\sum_{a=1}^{A} p(M_{0a}|data)} \Big/ \frac{\sum_{b=1}^{B} p(M_{1b})}{\sum_{a=1}^{A} p(M_{0a})},$$

which extend regular Bayes factors to multi-model settings (with the same interpretation–therefore the same abbreviation[4,11]).

Overview of the hypothesis testing approach

Prior to conducting this study, we have four confirmatory and two planned exploratory research questions that pertain to the broad study aims (see Table 1). Throughout the analysis, we perform a series of multiple two-group comparisons, comparing either the control and the consensus condition, the control and the updated consensus condition, or the consensus and the updated consensus condition. This also facilitates conclusions about both intervention messages separately. Our outcome variables can be split into two categories: 1) *continuous outcomes,* consisting of perceived scientific consensus (on the reality of climate change) and perceived scientific agreement (on climate change as a crisis), measured on a 0%–100% scale and 2) *ordinal outcomes,* consisting of belief in the reality of climate change, belief in the human causation of climate change, belief in climate change as a crisis, climate change worry, and support for public action measured on 7-point Likert scales. Since all continuous outcomes and all ordinal outcomes use the same model specifications, we describe the analytic plan for the hypotheses regarding continuous outcomes (i.e., consensus and agreement perception) jointly, and we do the same for all ordinal outcomes (i.e., belief in climate change, belief in human causation, belief in climate change as a crisis, climate change worry, and support for public action), in the corresponding sections (see Table 1 for mapping between hypotheses, group comparisons, and outcomes).

For consensus and agreement perception (i.e., continuous outcomes), we also conduct a moderation test to assess whether the intervention effect is modified by one of the moderators: familiarity with consensus/agreement messages, trust in climate scientists, and political ideology.

In all analyses, we will adjust for the following covariates: age (continuous), gender (categorical), education (dichotomized into no university degree vs. university degree), and political orientation (continuous). For all tests except the moderation by political ideology, we specify a directional alternative hypothesis.

Besides the specified hypotheses tests, we will report standardized mean differences between all groups for all outcomes (regardless of whether the outcome variable is continuous or ordinal), to facilitate incorporating the results into future meta-analyses. For moderation analyses, we will report standardized regression coefficients of the interaction effect. To make the results readily available for any future country-specific evidence synthesis, we will also report by-country specific standardized mean effect sizes and standardized regression coefficients in a supplementary table.

Bayes factor design analysis

We perform Bayes factor design analysis (BFDA) to evaluate the performance of our models[12,13]. In each BFDA, we examine model performance when simulating data from the null hypothesis of no effect and the alternative hypothesis assuming the presence of the effect. Under the presence of the effect, we consider two scenarios: a scenario based on previous findings (i.e., "empirical"), assuming the presence of the effect corresponding to effect sizes reported in previous meta-analyses[14,15] and a pessimistic scenario assuming the presence of an effect half the size.

In all scenarios, we further expand the BFDA set-up to examine the performance of the models under no heterogeneity, substantial heterogeneity, and excessive heterogeneity of the effect across countries (we always assume that countries differ in their baseline measurement, i.e., random intercepts). Consequently, our BFDA settings evaluate model performance across a wide range of conditions, including a robustness check for extremely unfavorable conditions (i.e., lower than expected effect size and excessive heterogeneity). In all BFDAs, we use an expected sample size of 10,000 participants spread equally across the three conditions and 25 countries. Consequently, each of the two-group comparisons is based on 6,666 participants. The number of participants and countries is based on resource and feasibility considerations (i.e., the data collection period is limited due to the collaborators' availability, and funding for data collection is currently limited to $1,000) and guided by previous experience in similar data collection efforts[16,17]. Due to computational constraints, each BFDA setting was reproduced 1,000 times, resulting in simulation error of the BFDA BFs classification estimates lower than 1.6%[10].

Analytic plan for the hypotheses

Continuous outcomes. This subsection describes hypothesis tests evaluating the effects of the classic and updated consensus message on consensus and agreement perceptions (H_{1a}, H_{2a}, and H_{2b}). Please refer to the "Continuous outcomes" section in Supplementary Materials on OSF (https://osf.io/udyvj?view_only=08f15cbdca5d41e7ad1c7fa159276c1) for details on the model parameterization and the Bayes factor design analysis.

Models
We will test H_{1a} (control vs. classic: consensus perceptions), H_{2a} (control vs. updated: consensus perceptions), and H_{2b} (control vs. updated: agreement perceptions) using Bayesian mixed-effects linear regression models, with participants at level 1 and countries at level 2. In all models, we will control for the pre-treatment measurement of the consensus/agreement perceptions. The hypothesis test will be performed by comparing two sets of models: a) models specifying the null hypothesis assuming the regression coefficient of the group difference is equal to 0 ($\beta_{group} = 0$) and b) models specifying the alternative hypothesis assuming the regression coefficient of the group difference is positive and distributed according to a prior distribution f ($\beta_{group} \sim f()$). Each model set, i.e., models a and b, is represented via two models: 1) a model assuming no differences across countries in the group effect ($\tau_{group} = 0$; random intercept only model) and 2) a model assuming differences across countries in the group effect ($\tau_{group} = g()$; random intercept and random slope model).

We will estimate the models using the *BayesFactor* R package[18]. We will use a set prior scale of $r = 0.50$ for the fixed-effects regression coefficients, the "medium" prior scale closely corresponding to the previously reported meta-analytic effect of $g = 0.55$[14] and $r = 0.25$ for the random-effects regression coefficients, assuming that the between-country variability will be approximately half the effect size. The common intercept and residual variance use the default Jeffreys prior.

Hypothesis test
Each hypothesis is then evaluated by comparing the alternative hypothesis models (i.e., positive group difference) without (M_{b1}) and with (M_{b2}) between-country heterogeneity to the null hypothesis models (i.e., no group difference) without (M_{a1}) and with (M_{a2}) between-country heterogeneity using inclusion Bayes factors.

Bayes factor design analysis
We used standardized mean differences (Cohen's *d*) to define the simulation settings for continuous outcomes. We simulated data under the null hypothesis of no effect ($d = 0$), an empirical alternative hypothesis ($d = 0.50$; a slightly lower effect size than was reported in a recent meta-analysis: Hedges' $g = 0.55$[14]), and a pessimistic alternative hypothesis ($d = 0.25$; half the effect size of the empirical alternative hypothesis). For heterogeneity in effect sizes, we considered no heterogeneity ($\tau_{group} = 0$), substantial heterogeneity assuming the standard deviation of true effects is half the effect size ($\tau_{group} = 0.25$), i.e., the maximum amount of heterogeneity we could observe while keeping approximately 97.5% of the country specific effects still positive[1], and extreme heterogeneity assuming the standard deviation of true effects equals the effect size ($\tau_{group} = 0.50$), i.e., almost 15.9% of the effect sizes are in the opposite direction than the mean. We based auxiliary simulation parameters such as the pre-treatment means, pre- and post- treatment residual variances, and pre-post treatment correlations on a pilot study (see Supplementary Information B).

Supplementary Fig. 2 visualizes the proportion of Bayes factors resulting in strong evidence for the alternative hypothesis under the BFDA conditions of primary interests: the null and

[1] These are very conservative settings, given that the previously mentioned meta-analysis reported heterogeneity estimate 1.8 times lower ($\tau = 0.139$)[24].

alternative hypotheses under the no and substantial heterogeneity. We verify that the specified tests provide strong evidence $BF_{10} > 10$ for the alternative hypothesis in both heterogeneity conditions under the alternative hypothesis (in all cases under no heterogeneity and in 99.4% of cases under substantial heterogeneity). Furthermore, the specified tests provide strong evidence for the null hypothesis $BF_{10} < 1/10$ in 90.9% of the no heterogeneity condition. It should be noted that, in case of substantial heterogeneity, inferences about the absence of the effect are more challenging, and the test can provide strong evidence for the absence of the effect only in the minority of the cases (5.0%).

The robustness check under unfavorable conditions, i.e., half of the expected effect size, excessive heterogeneity, or both, show that while the specified tests are considerably less powerful, they rarely produce misleading evidence. We almost never find misleading evidence for the null hypothesis ($BF_{10} < 1/10$; max. 0.3% under pessimistic alternative hypothesis with excessive heterogeneity) or misleading evidence for the alternative hypothesis ($BF_{10} > 10$, max. 0.8% under null hypothesis with excessive heterogeneity). See Table 2 in the Supplementary Materials on OSF for detailed performance estimates under each condition.

Supplementary Fig. 3: Results of Bayes Factor Design Analysis for Continuous Outcomes.

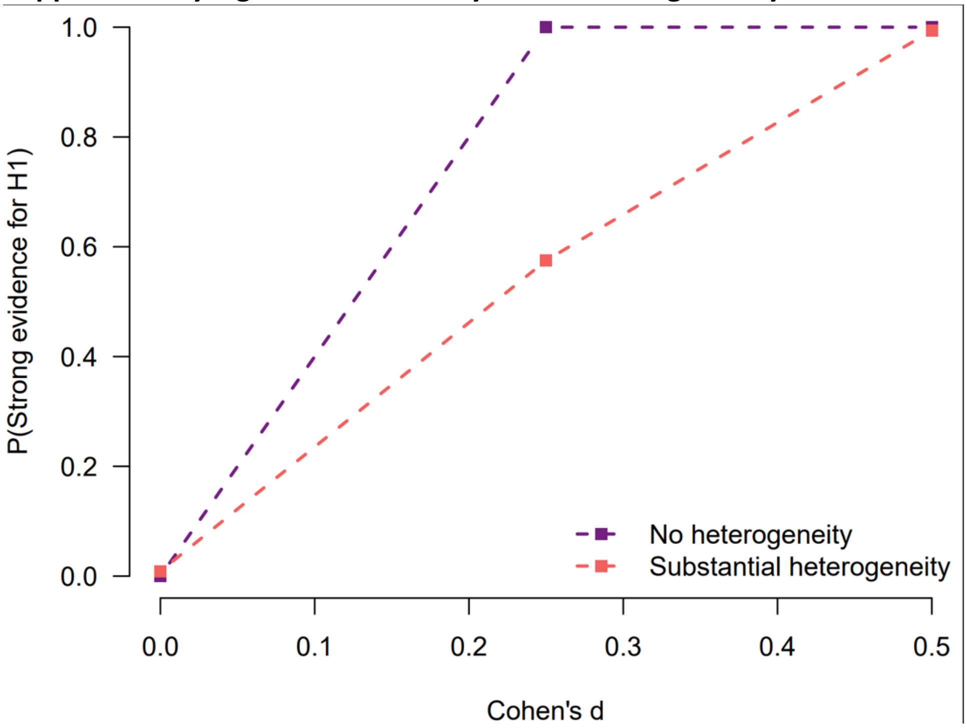

Note. The proportion of strong evidence ($BF_{10} > 10$; *y*-axis) across different effect sizes *(x*-axis) and between-country heterogeneity of the effect sizes ("No" = countries differ only in the baseline measure, "Substantial" = countries differ in both the baseline and effect sizes). Results are based on one-sided inclusion Bayes factors from Bayesian model-averaged mixed-effects linear regressions, with no multiple comparison adjustment.

Ordinal outcomes. This subsection describes hypothesis tests evaluating the effects of the classic and updated consensus messages on personal beliefs in the reality of climate change (H_{1b} and H_{2c}), human causation of climate change (H_{1c} and H_{2d}), climate change as a crisis (H_{2e} and H_{3a}), worry (H_{1d}, H_{2f}, and H_{3b}), and support for public action (H_{1e}, H_{2g}, and H_{3c}). Please refer to the "Ordinal outcomes" section in the Supplementary Materials on OSF for details on the model parameterization and the Bayes factor design analysis.

Models

We will test the hypotheses using Bayesian mixed-effects cumulative probit regression models, with participants at level 1 and countries at level 2. We use the ordinal hypothesis testing framework[19] that allows us to distinguish between different shifts in the ordinal scale response patterns (no, random, constant, and dominant). The hypothesis test will be performed by comparing two sets of models: a) models specifying the null hypothesis assuming the response pattern is either not affected by the treatment or that the response pattern is only randomly affected by the treatment, and b) models specifying the alternative hypothesis assuming the response pattern is either constantly positively affected by the treatment or dominantly positively affected by the treatment. Each model set, i.e., models a and b, is again represented via two models: 1) a model assuming no differences across countries in the group effect ($\tau_{group} = 0$; random intercept only model) and 2) a model assuming differences across countries in the group effect ($\tau_{group} = g()$; random intercept and random slope model).

We will estimate the models using *Stan*[20] and the *Rstan* R package[21] and compute the marginal likelihood via bridge sampling using the *bridgesampling* R package[22]. Based on recent meta-analyses[14,15], we expect smaller effects of the interventions on the ordinal outcomes than on the continuous outcomes (approximately Cohen's $d = 0.10$). When simulating from models corresponding to the alternative hypotheses, we found that a mean shift in the latent variable $\theta = 0.14$ converts to the Cohen's d of approximately 0.10 (if the data were analyzed as continuous). Therefore, we set the standard deviation of the normal prior distribution on group difference in the standard normal latent ability to $\theta = 0.14$ and the standard deviation of the normal distribution for the random effects to $\tau = 0.07$, again assuming that the between-country variability will be approximately half the effect size. The common thresholds use the default standard normal prior distribution[19].

Hypothesis test

Each hypothesis is then evaluated by comparing the alternative hypothesis models (i.e., positive constant shift differences) without (M_{b1}) and with (M_{b2}) between-country heterogeneity and positive dominant shift differences without (M_{b3}) and with (M_{b4}) between-country heterogeneity to the null hypothesis models (i.e., no differences) without (M_{a1}) and with (M_{a2}) between-country heterogeneity and random shift differences without (M_{a3}) and with (M_{a4}) between-country heterogeneity, using inclusion Bayes factors.

Bayes factor design analysis

We used mean differences in the standard normal latent variable (θ) to define the simulation settings for ordinal outcomes. We simulated data under the null hypothesis of no effect ($\theta = 0$), an empirical alternative hypothesis ($\theta = 0.14$; corresponding to an effect size that was reported in recent meta-analyses: $d = 0.10$[14,15]), and a pessimistic alternative hypothesis ($\theta = 0.07$; half the effect size of the empirical alternative hypothesis). For heterogeneity in effect sizes, we follow the same logic as for the continuous outcomes and consider no heterogeneity ($\tau_{group} = 0$), substantial heterogeneity assuming the standard deviation of true effects is half the effect size ($\tau_{group} = 0.07$), and extreme heterogeneity assuming the standard deviation of true effects equals the effect size ($\tau_{group} = 0.14$). We specified two common response patterns to verify the performance of our tests under different settings, a centered response pattern where the most common response is in the center of the 7-point Likert scale and a skewed response pattern where the most common response is at the upper bound of the 7-point Likert scale (representing ceiling effects). Since the results did not meaningfully differ between the response patterns, we report the aggregated results.

Supplementary Fig. 3 depicts the proportion of Bayes factors resulting in strong evidence for the alternative hypothesis under the BFDA conditions of primary interests: the null and alternative hypotheses under the no and substantial heterogeneity. We verify that the specified tests provide strong evidence $BF_{10} > 10$ for the alternative hypothesis in majority of the cases in both heterogeneity conditions under the alternative hypothesis (in 90.0% of cases under no heterogeneity and in 86.6% of cases under substantial heterogeneity). Furthermore, the specified tests also often provide strong evidence for the null hypothesis $BF_{10} < 1/10$ (in 51.3% of the no heterogeneity condition and 48.7% cases under substantial heterogeneity). Please refer to Table 5 in the Supplementary Materials on OSF for detailed performance estimates under each condition.

The robustness check under unfavorable conditions (i.e., half of the expected effect size, excessive heterogeneity, or both), shows that the specified tests are less powerful in the substantial or excessive heterogeneity conditions under the pessimistic alternative hypothesis. However, we rarely find misleading evidence for the null hypothesis ($BF_{10} < 1/10$; max. 0.8% under empirical alternative hypothesis with excessive heterogeneity) or misleading evidence for the alternative hypothesis ($BF_{10} > 10$, max. 3.1% under null hypothesis with excessive heterogeneity).

Supplementary Fig. 4: Results of Bayes Factor Design Analysis for Ordinal Outcomes.

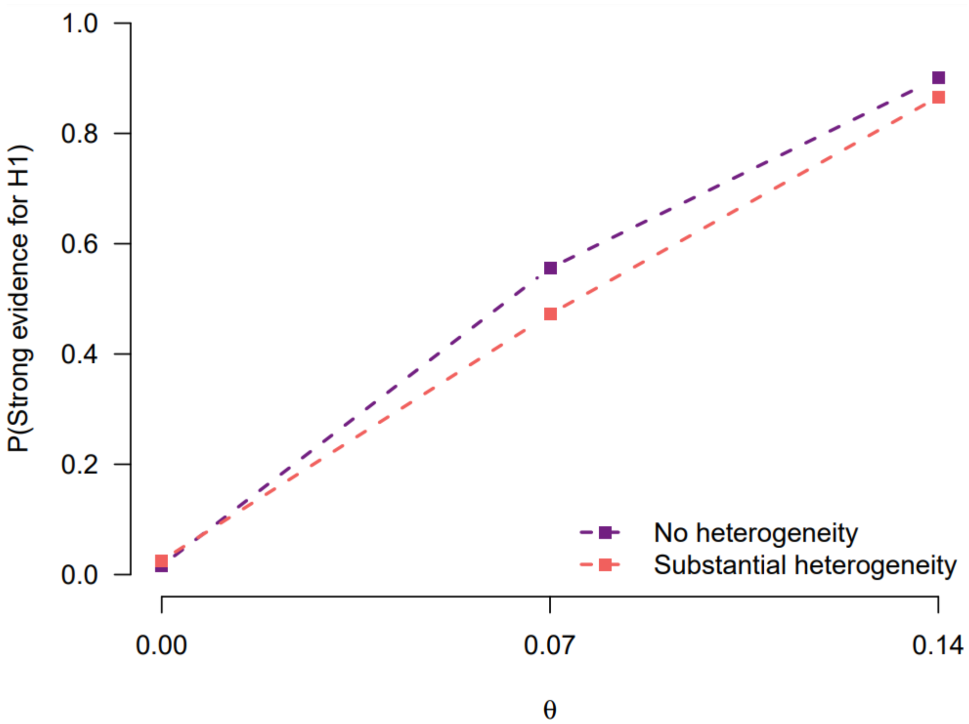

Note. The proportion of strong evidence ($BF_{10} > 10$; *y*-axis) across different effect sizes (*x*-axis) and between-country heterogeneity of the effect sizes ("No" = countries differ only in the baseline measure, "Substantial" = countries differ in both the baseline and effect sizes). Results are based on one-sided inclusion Bayes factors from Bayesian model-averaged mixed-effects cumulative probit regression, with no multiple comparison adjustment.

Moderation of continuous outcomes. This subsection describes the hypothesis tests evaluating whether the effectiveness of the classic consensus intervention in changing consensus perceptions is moderated by message familiarity (H_{4a}), trust in climate scientists (H_{4b}), as well as political ideology (H_{4c}). Please refer to the “Moderation” subsection of the “Continuous outcomes” section in the Supplementary Materials on OSF for details on the parameterization of the models and the Bayes factor design analysis.

Models

We will test the hypotheses by extending the Bayesian mixed-effects linear regression models described in the “Continuous outcomes” section with the given moderator and its interaction with the group effect. The hypothesis test will be performed by comparing two sets of models: a) models specifying the null hypothesis assuming the regression coefficient

of the interaction between the moderator and the group effect is equal to 0 ($\beta_{moderator*treatment}$ = 0) and b) models specifying the alternative hypothesis assuming the regression coefficient of the interaction between the moderator and the group effect is positive and distributed according to a prior distribution f ($\beta_{moderator*group} \sim f()$). Each model set, i.e., models a and b, is represented via two models: 1) a model assuming the absence of the moderator's main effect and 2) a model assuming the presence of the moderator's main effect.

We will estimate the models using the *BayesFactor* R package[18] with the same prior distribution settings as in the "Continuous outcomes" section.

Hypothesis test
Each hypothesis is then evaluated by comparing all versions of the alternative hypothesis models (i.e., positive interaction between the moderator and group effect), M_{b1} (no moderator main effect) and M_{b2} (moderator main effect), to all versions of the null hypothesis models (i.e., no interaction between the moderator and group effect), M_{a1} (no moderator main effect) and M_{a2} (moderator main effect), using inclusion Bayes factors.

Bayes factor design analysis
We used standardized regression coefficients (β) to define the simulation settings for continuous outcomes. We simulated data under the null hypothesis of no effect (β = 0), a best guess alternative hypothesis (β = 0.30; our best guess of the possible moderation effect – 60% of the main effect of the group), and a pessimistic alternative hypothesis (β = 0.15; half the effect size of the best guess alternative hypothesis). For heterogeneity in effect sizes, we again follow the same logic as in the continuous outcomes, we considered no heterogeneity assuming the standard deviation of true effects is half the effect size ($\tau_{moderator*treatment}$ = 0), substantial heterogeneity ($\tau_{moderator*treatment}$ = 0.15), and extreme heterogeneity assuming the standard deviation of true effects equals the effect size ($\tau_{moderator*treatment}$ = 0.30). For computational feasibility, we simulated only from the alternative model assuming the presence of the best guess alternative hypothesis and substantial heterogeneity in the effect. Again, we based auxiliary simulation parameters, such as the pre-treatment means, pre- and post-treatment residual variances, as well as pre-post-treatment correlations on the pilot data (see Supplementary Information B).

Supplementary Fig. 4 visualizes the proportion of Bayes factors resulting in strong evidence for the alternative hypothesis under the BFDA conditions of primary interests: the null and alternative hypotheses under no and substantial heterogeneity. We verify that the specified tests provide strong evidence $BF_{10} > 10$ for the alternative hypothesis in both heterogeneity conditions under the alternative hypothesis (in all cases under no heterogeneity and in 99.7% of cases under substantial heterogeneity). Furthermore, the specified tests also provide strong evidence for the null hypothesis $BF_{10} < 1/10$ (in 95.6% of the no heterogeneity condition and 91.6% cases under substantial heterogeneity). See Table 4 in the Supplementary Materials on OSF for detailed performance estimates under each condition.

The robustness check under unfavorable conditions (i.e., smaller than expected effect size, excessive heterogeneity, or both), shows that the specified tests do not suffer much in the

excessive heterogeneity condition under either the null or the best guess alternative hypothesis. The largest decrease in power is under the pessimistic alternative hypothesis, yet they can provide strong evidence in approximately 55% of cases. The tests rarely find misleading evidence: In fewer than 5% of cases, we find misleading evidence in favor of the null hypothesis in all but under the pessimistic alternative hypothesis and excessive heterogeneity (13.2%), and in only 2.4% of cases we find misleading evidence in favor of the alternative hypothesis under the presence of excessive heterogeneity.

Supplementary Fig. 5: Results of Bayes Factor Design Analysis for Moderation of Continuous Outcomes.

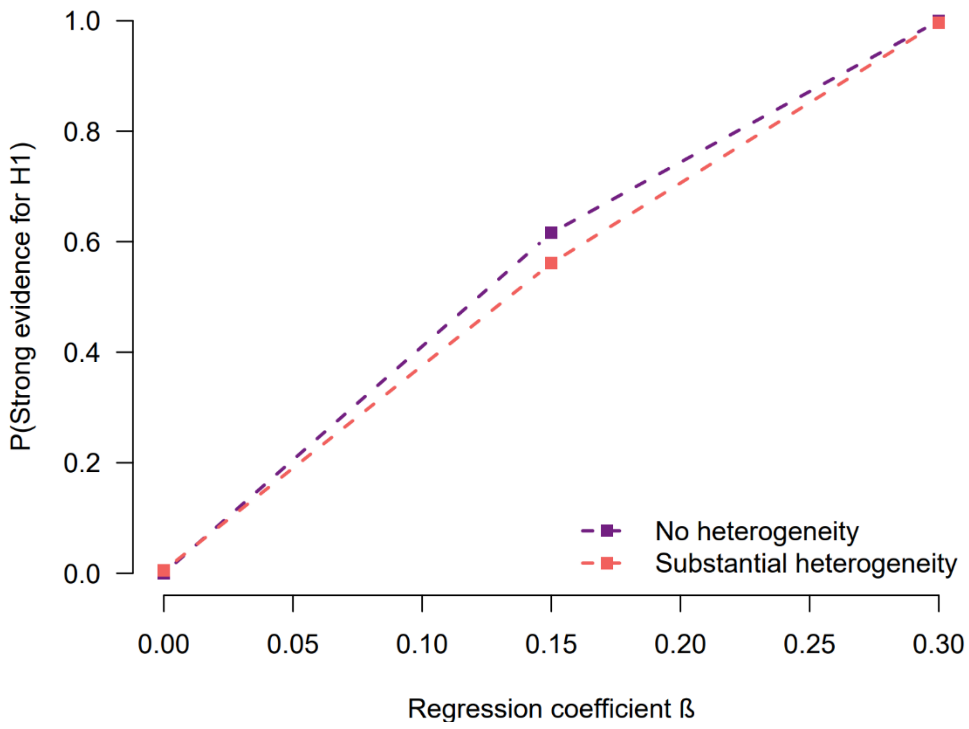

Note. The proportion of strong evidence ($BF_{10} > 10$; *y*-axis) across different effect sizes of the interactions of moderator and group membership (*x*-axis) and between-country heterogeneity of the effect sizes ("No" = countries differ only in the baseline measure, "Substantial" = countries differ in both the baseline and effect sizes of the interactions of moderator and group membership). Results are based on one-sided inclusion Bayes factors from Bayesian model-averaged mixed-effects linear regressions, with no multiple comparison adjustment.

Planned exploratory analyses

Between-country heterogeneity

We will perform follow-up exploratory analyses of the between-country heterogeneity in group effects specified in the “Continuous outcomes” and “Ordinal outcomes” sections (i.e., $H_{1a\text{-}e}$, $H_{2a\text{-}h}$, and $H_{3a\text{-}c}$). We use the same models as specified in the aforementioned sections; however, we group the models into different sets to perform an inclusion Bayes factor test for the presence vs. absence of between-county heterogeneity (BF_{rf}) in the group effects.

The alternative hypothesis of the presence of between-county heterogeneity in the group effects is represented via all models assuming the presence of between-county

heterogeneity ($\tau_{group} = g()$) regardless of whether the effect is present or not in continuous outcomes, or the assumed response pattern change in ordinal outcomes. The null hypothesis of the absence of the between-county heterogeneity in the group effects is represented via all models assuming the absence of the between-county heterogeneity ($\tau_{group} = 0$) regardless of whether the effect is present or not in continuous outcomes, or the assumed response pattern change in ordinal outcomes. Please refer to the corresponding section in the Supplementary Materials on OSF for details on model parameterizations.

Supplementary Fig. 5A visualizes the proportion of Bayes factors resulting in strong evidence either for or against the presence of the between-country heterogeneity in group differences of continuous outcomes aggregated across the alternative hypotheses about the presence vs. absence of the effect. We find that the specified tests always provide strong evidence $BF_{rf} > 10$ for the presence of heterogeneity in both heterogeneity conditions. Furthermore, the specified tests also provide strong evidence for the absence of heterogeneity $BF_{rf} < 1/10$ in 60.2% of the cases when heterogeneity is absent (although they provide misleading strong evidence for the presence of heterogeneity in 12.6% of such cases). See Table 3 in the Supplementary Materials on OSF for detailed performance estimates under each condition.

Supplementary Fig. 5B visualizes the proportion of Bayes factors resulting in strong evidence either for or against the presence of the between-country heterogeneity in group differences of ordinal outcomes aggregated across the alternative hypotheses about the presence vs. absence of the effect. We find that the specified tests provide strong evidence $BF_{rf} > 10$ for the presence of heterogeneity in only 18.0% of cases under substantial heterogeneity and in 90.6% of cases under extreme heterogeneity condition. Furthermore, the specified tests also provide strong evidence for the absence of heterogeneity $BF_{rf} < 1/10$ in 47.8% of the cases when heterogeneity is absent. The rate of misleading evidence for either the absence or presence of heterogeneity is low (3.2% and 0.4%, respectively). See Table 6 in the Supplementary Materials on OSF for detailed performance estimates under each condition.

Supplementary Fig. 6A: Results of Bayes Factor Design Analysis for Heterogeneity in Continuous Outcomes.

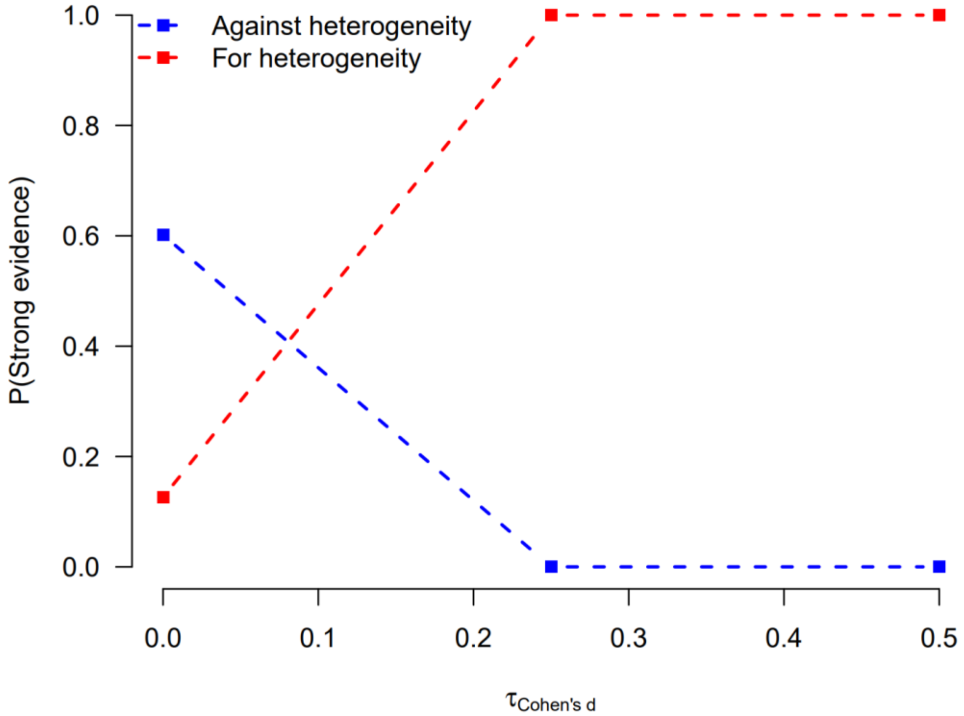

Note. The proportion of strong evidence (BF_{rf} > 10 or BF_{rf} > 1/10; *y*-axis) either against or for the presence of between-country heterogeneity in group differences (blue and red lines, respectively) aggregated across the alternative hypotheses about the presence vs. absence of the effect. Results are based on one-sided inclusion Bayes factors from Bayesian model-averaged mixed-effects linear regressions, with no multiple comparison adjustments.

Supplementary Fig. 6B: Results of Bayes Factor Design Analysis for Heterogeneity in Ordinal Outcomes.

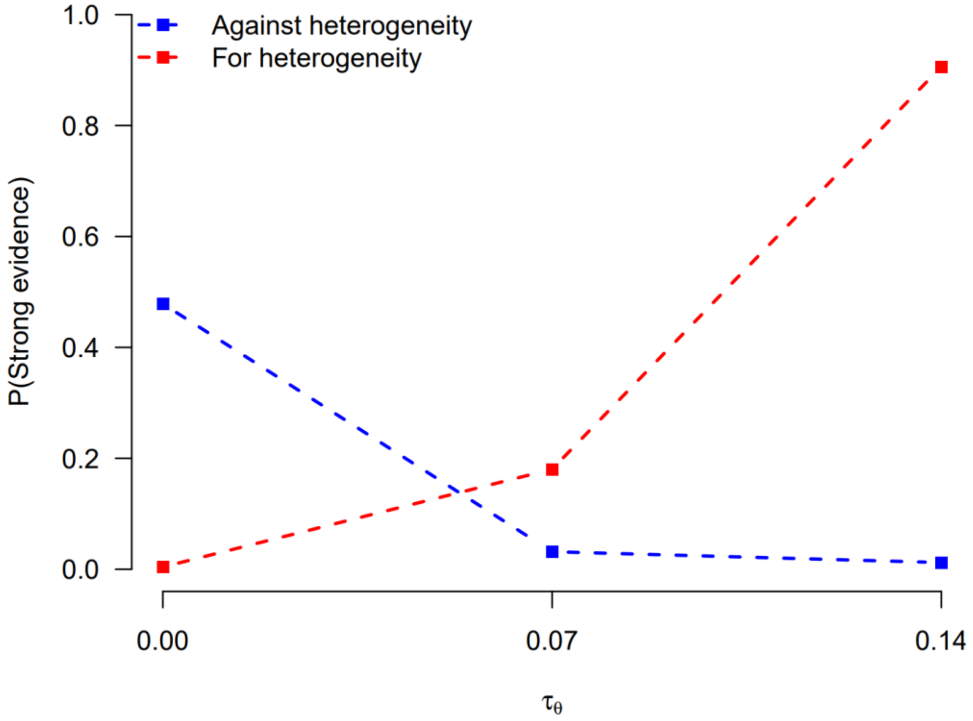

Note. The proportion of strong evidence (BF_{rf} > 10 or BF_{rf} > 1/10; *y*-axis) either against or for the presence of between-country heterogeneity in group differences (blue and red lines, respectively) aggregated across the alternative hypotheses about the presence vs. absence of the effect. Results are based on one-sided inclusion Bayes factors from Bayesian model-averaged mixed-effects cumulative probit regressions, with no multiple comparison adjustments.

4. References

1. Tollefson, J. Top climate scientists are sceptical that nations will rein in global warming. *Nature* **599**, 22–24 (2021).
2. Myers, T. A., Maibach, E., Peters, E. & Leiserowitz, A. Simple Messages Help Set the Record Straight about Scientific Agreement on Human-Caused Climate Change: The Results of Two Experiments. *PLOS ONE* **10**, e0120985 (2015).
3. Jeffreys, H. Some Tests of Significance, Treated by the Theory of Probability. *Proceedings of the Cambridge Philosophy Society* **31**, 203–222 (1935).
4. Hinne, M., Gronau, Q. F., van den Bergh, D. & Wagenmakers, E.-J. A conceptual introduction to Bayesian model averaging. *Advances in Methods and Practices in Psychological Science* **3**, 200–215 (2020).
5. Hoeting, J. A., Madigan, D., Raftery, A. E. & Volinsky, C. T. Bayesian model averaging: a tutorial. *Statistical Science* **14**, 382–401 (1999).
6. Rouder, J. N. & Morey, R. D. Teaching Bayes' theorem: Strength of evidence as predictive accuracy. *The American Statistician* **73**, 186–190 (2019).
7. Etz, A. & Wagenmakers, E.-J. J. B. S. Haldane's contribution to the Bayes factor hypothesis Test. *Statistical Science* **32**, 313–329 (2017).
8. Lee, M. D. & Wagenmakers, E.-J. *Bayesian cognitive modeling: A practical course*. (Cambridge University Press, 2013).
9. Jeffreys, H. *Theory of probability*. (Oxford University Press, 1939).
10. Morris, T. P., White, I. R. & Crowther, M. J. Using simulation studies to evaluate statistical methods. *Statistics in Medicine* **38**, 2074–2102 (2019).
11. Gronau, Q. F., Heck, D. W., Berkhout, S. W., Haaf, J. M. & Wagenmakers, E.-J. A primer on Bayesian model-averaged meta-analysis. *Advances in Methods and Practices in Psychological Science* **4**, 1–19 (2021).
12. Schönbrodt, F. D. & Wagenmakers, E.-J. Bayes factor design analysis: Planning for compelling evidence. *Psychonomic Bulletin & Review* **25**, 128–142 (2018).
13. Stefan, A. M., Gronau, Q. F., Schönbrodt, F. D. & Wagenmakers, E.-J. A tutorial on Bayes factor design analysis using an informed prior. *Behavior Research Methods* **51**, 1042–1058 (2019).
14. van Stekelenburg, A., Schaap, G., Veling, H., van 't Riet, J. & Buijzen, M. Scientific-Consensus Communication About Contested Science: A Preregistered Meta-Analysis. *Psychol Sci* 9567976221083220 (2022) doi:10.1177/09567976221083219.
15. Rode, J. B. *et al.* Influencing climate change attitudes in the United States: A systematic review and meta-analysis. *Journal of Environmental Psychology* **76**, 101623 (2021).
16. Ruggeri, K. *et al.* The globalizability of temporal discounting. *Nature Human Behaviour* **6**, 1386–1397 (2022).
17. Ruggeri, K. *et al.* The general fault in our fault lines. *Nature Human Behaviour* (2021) doi:10.1038/s41562-021-01092-x.

18. Morey, R. D., Rouder, J. N., Jamil, T. & Morey, M. R. D. Package ‘bayesfactor’. *URLh http://cran/r-projectorg/web/packages/BayesFactor/BayesFactor pdf i (accessed 1006 15)* (2015).
19. Schnuerch, M., Haaf, J. M., Sarafoglou, A. & Rouder, J. N. Meaningful comparisons with ordinal-scale items. *Collabra: Psychology* **8**, 38594 (2022).
20. Carpenter, B. *et al.* Stan: A probabilistic programming language. *Journal of statistical software* **76**, (2017).
21. Stan Development Team. RStan: the R interface to Stan. (2020).
22. Gronau, Q. F., Singmann, H. & Wagenmakers, E.-J. bridgesampling: An R Package for Estimating Normalizing Constants. *Journal of Statistical Software* **92**, 1–29 (2020).
