# Supplementary material for: A 27-country test of communicating the scientific consensus on climate change
Source: Nat Hum Behav. 2024 Aug 26;8(10):1892–905. doi: 10.1038/s41562-024-01928-2 (PMC11493676; doi:10.1038/s41562-024-01928-2)
Supplement: Supplementary file 2 — Reporting Summary [file 41562_2024_1928_MOESM2_ESM.pdf]

nature portfolio

Corresponding author(s): Geiger & Ruggeri

Last updated by author(s): Feb 28, 2024

Reporting Summary

Nature Portfolio wishes to improve the reproducibility of the work that we publish. This form provides structure for consistency and transparency in reporting. For further information on Nature Portfolio policies, see our Editorial Policies and the Editorial Policy Checklist.

Statistics

For all statistical analyses, confirm that the following items are present in the figure legend, table legend, main text, or Methods section.

n/a	Confirmed	
☐	☒	The exact sample size (*n*) for each experimental group/condition, given as a discrete number and unit of measurement
☐	☒	A statement on whether measurements were taken from distinct samples or whether the same sample was measured repeatedly
☐	☒	The statistical test(s) used AND whether they are one- or two-sided<br>*Only common tests should be described solely by name; describe more complex techniques in the Methods section.*
☐	☒	A description of all covariates tested
☐	☒	A description of any assumptions or corrections, such as tests of normality and adjustment for multiple comparisons
☐	☒	A full description of the statistical parameters including central tendency (e.g. means) or other basic estimates (e.g. regression coefficient) AND variation (e.g. standard deviation) or associated estimates of uncertainty (e.g. confidence intervals)
☐	☒	For null hypothesis testing, the test statistic (e.g. *F*, *t*, *r*) with confidence intervals, effect sizes, degrees of freedom and *P* value noted<br>*Give P values as exact values whenever suitable.*
☐	☒	For Bayesian analysis, information on the choice of priors and Markov chain Monte Carlo settings
☐	☒	For hierarchical and complex designs, identification of the appropriate level for tests and full reporting of outcomes
☐	☒	Estimates of effect sizes (e.g. Cohen's *d*, Pearson's *r*), indicating how they were calculated

Our web collection on statistics for biologists contains articles on many of the points above.

Software and code

Policy information about availability of computer code

Data collection	Data collection was conducted using the Qualtrics XM web service platform (version: July/August 2023).
Data analysis	The R code for the main analyse and the Bayesian Design analyses are publicly available on the Open Science Framework (https://osf.io/z6quh/). To run the mixed-effects linear regression models, we used the R package BayesFactor. For the mixed-effects cumulative probit regression models, we used Stan and the Rstan R package, and computed the marginal likelihood via bridge sampling using the bridgesampling R package.

For manuscripts utilizing custom algorithms or software that are central to the research but not yet described in published literature, software must be made available to editors and reviewers. We strongly encourage code deposition in a community repository (e.g. GitHub). See the Nature Portfolio guidelines for submitting code & software for further information.

Data

Policy information about availability of data

All manuscripts must include a data availability statement. This statement should provide the following information, where applicable:

- Accession codes, unique identifiers, or web links for publicly available datasets
- A description of any restrictions on data availability
- For clinical datasets or third party data, please ensure that the statement adheres to our policy

The raw and cleaned datasets for all analyses reported in this manuscript are publicly available under a CC-By Attribution International 4.0 license on the Open Science Framework (https://osf.io/z6quh/).

Research involving human participants, their data, or biological material

Policy information about studies with human participants or human data. See also policy information about sex, gender (identity/presentation), and sexual orientation and race, ethnicity and racism.

Reporting on sex and gender	Gender was considered as demographic information and as a covariate in the current study design. Gender was determined based on self-reporting ("What is your gender?", including male, female, other, and prefer not to say as response options). The source data include disaggregated gender data, and consent has been obtained for sharing these individual-level data. Overall, our sample included 57% participants that identified as female, 42% as male, and 1% as other.
Reporting on race, ethnicity, or other socially relevant groupings	We assessed ethnicity in our survey to describe our samples. Ethnicity was determined based on self-reporting ("Please choose which best describes you."), with response categories carefully adapted to the country-specific context. Due to the plurality of the overall response options, which makes it impossible to provide a single summary statistic, we did not use this item to describe the samples.
Population characteristics	Participants were 57% female, with a mean age of 33.7 (SD = 13.3). Almost 100% of participants had completed some formal education, with 68.1% completing a university degree and 32.7% were current students. Most participants (81.6%) lived in urban areas.
Recruitment	We use what we refer to as the Demic-Veckalov (named for Emir Demic and Bojana Veckalov) method for sampling: All collaborators used a range of circulation points, including email lists, discussion boards, and social media pages to recruit as random a sample as possible. This meant we primarily did not use individual pages to recruit, but instead, found recent posts with high engagement (often related to popular media topics except climate change/sustainability) as well as common interest platforms (e.g., Reddit channels). We also contacted universities and other organizations to assist with circulation. The primary forms of bias that this could create would be over-representation of individuals with computers/social media accounts, younger and more educated participants (due to the types of news stories often used as a conduit for recruiting), and individuals that speak the primary local language.
Ethics oversight	We obtained ethical approval from the Institutional Review Board from the University of Amsterdam (the Netherlands; protocol FMG-1123) and the University of Porto (Portugal; protocol 2023/06-12).

Note that full information on the approval of the study protocol must also be provided in the manuscript.

Field-specific reporting

Please select the one below that is the best fit for your research. If you are not sure, read the appropriate sections before making your selection.

☐ Life sciences ☒ Behavioural & social sciences ☐ Ecological, evolutionary & environmental sciences

For a reference copy of the document with all sections, see nature.com/documents/nr-reporting-summary-flat.pdf

Behavioural & social sciences study design

All studies must disclose on these points even when the disclosure is negative.

Study description	A 27-country study testing the effectiveness of scientific consensus messaging on climate change for climate change beliefs, worry, and support for public action. All participants completed an approximately 5-min survey which randomly assigned participants to one of three messages: a control message (97% of dentists recommend brushing your teeth twice per day), the classic scientific consensus message (97% of climate scientists agree that human-caused climate change is happening), and an updated scientific consensus message that additionally presented the agreement among climate scientists that climate change is a crisis (In addition, 88% of climate scientists agree that climate change constitutes a crisis).
Research sample	Convenience sample of adults (18 years and older) from 27 countries (57% female; mean age = 33). Samples were not recruited in a way that ensured representativeness, but instead we focused on obtaining a large enough sample from a diverse set of countries that provides sufficiently powered estimates for comparisons across and partly between countries.

Sampling strategy	We used the Demić–Većkalov method (Ruggeri et al., 2022) to obtain convenience samples, as described above under "Recruitment". The overall and per-country sample size was determined based on Bayes Factor Design Analyses (BFDA). Details on the BFDA can be found in the Supplementary Materials 3.5.<br><br>Reference: Ruggeri, K., Panin, A., Vdovic, M., Većkalov, B., Abdul-Salaam, N., Achterberg, J., ... & Toscano, F. (2022). The globalizability of temporal discounting. Nature Human Behaviour, 6(10), 1386-1397.
Data collection	All participants completed the study via Qualtrics; no researcher was present at the time of data collection and there were no conditions for blinding. Participants completed the survey in the primary local langauge (in some cases, an additional English version of the survey was offered).
Timing	All data were collected between late July 27 and August 4, 2023.
Data exclusions	At the participant level, we excluded respondents who failed the attention check (n = 1,032) or finished the survey in less than 2 minutes (n = 0; based on pilot data in which none of the respondents completed a very similar survey in less than 2 minutes). In addition, for paid samples, responses identified as potential bots (Qualtrics variable: Q_RecaptchaScore < .50; n = 3), duplicates (Qualtrics variable: Q_RelevantIDDuplicateScore ≥ 75; n = 9), or fraudulent (Qualtrics variable: Q_RelevantIDFraudScore ≥ 30, as per Qualtrics recommendations; n = 6) were excluded. Given that these are not equally functional and thus informative across countries, we kept respondents for whom these metrics were not recorded. All exclusion criteria were preregistered.<br><br>Participants with missing data were excluded on an analysis-by-analysis basis. Prior to data collection, we expected few missing values due to the forced-response format of all items. This was confirmed, with 130 missing values on belief in the human causation of climate change and 128 missing values on gender.<br><br>We did not define or remove any outliers, as all measures are bounded, which effectively guards us from any outliers.
Non-participation	Out of 21,463 people who clicked on the link, 11,702 participants completed the survey. Out of these, 10,527 participants remained after further exclusions (see Data exclusions above).
Randomization	Participants were randomly assigned to one of three groups (control, classic scientific consensus, and updated scientific consensus condition). This randomization was implemented via Qualtrics, such that each message was equally often presented across all participants.

Reporting for specific materials, systems and methods

We require information from authors about some types of materials, experimental systems and methods used in many studies. Here, indicate whether each material, system or method listed is relevant to your study. If you are not sure if a list item applies to your research, read the appropriate section before selecting a response.

Materials & experimental systems

n/a	Involved in the study
☒	☐ Antibodies
☒	☐ Eukaryotic cell lines
☒	☐ Palaeontology and archaeology
☒	☐ Animals and other organisms
☒	☐ Clinical data
☒	☐ Dual use research of concern
☒	☐ Plants

Methods

n/a	Involved in the study
☒	☐ ChIP-seq
☒	☐ Flow cytometry
☒	☐ MRI-based neuroimaging
